# Serum rheumatoid factor IgA, anti-citrullinated peptide antibodies with secretory components, and anti-carbamylated protein antibodies associate with interstitial lung disease in rheumatoid arthritis

**DOI:** 10.1186/s12891-021-04985-0

**Published:** 2022-01-13

**Authors:** Shomi Oka, Takashi Higuchi, Hiroshi Furukawa, Kota Shimada, Akira Okamoto, Atsushi Hashimoto, Akiko Komiya, Koichiro Saisho, Norie Yoshikawa, Masao Katayama, Toshihiro Matsui, Naoshi Fukui, Kiyoshi Migita, Shigeto Tohma

**Affiliations:** 1 Department of Rheumatology, National Hospital Organization Tokyo National Hospital, 3-1-1 Takeoka, Kiyose, 204-8585 Japan; 2 Clinical Research Center for Allergy and Rheumatology, National Hospital Organization Sagamihara National Hospital, 18-1 Sakuradai, Minami-ku, Sagamihara, 252-0392 Japan; 3 Department of Nephrology, Ushiku Aiwa General Hospital, 896 Shishiko-cho, Ushiku, 300-1296 Japan; 4 Department of Rheumatology, National Hospital Organization Sagamihara National Hospital, 18-1 Sakuradai, Minami-ku, Sagamihara, 252-0392 Japan; 5 Department of Rheumatic Diseases, Tokyo Metropolitan Tama Medical Center, 2-8-29 Musashi-dai, Fuchu, 183-8524 Japan; 6 Department of Rheumatology, National Hospital Organization Himeji Medical Center, 68 Hon-machi, Himeji, 670-8520 Japan; 7 Department of Internal Medicine, Sagami Seikyou Hospital, 6-2-11 Sagamiohno, Minami-ku, Sagamihara, 252-0303 Japan; 8 Department of Clinical Laboratory, National Hospital Organization Sagamihara National Hospital, 18-1 Sakuradai, Minami-ku, Sagamihara, 252-0392 Japan; 9 Department of Orthopedics/Rheumatology, National Hospital Organization Miyakonojo Medical Center, 5033-1 Iwayoshi-cho, Miyakonojo, 885-0014 Japan; 10 Tanimura Hospital, 10-2 Kitakoji, Nobeoka, 882-0041 Japan; 11 Department of Internal Medicine, National Hospital Organization Nagoya Medical Center, 4-1-1 Sannomaru, Naka-ku, Nagoya, 460-0001 Japan; 12 Clinical Research Center, National Hospital Organization Nagasaki Medical Center, 2-1001-1 Kubara, Omura, 856-8562 Japan; 13 Department of Gastroenterology and Rheumatology, Fukushima Medical University School of Medicine, 1 Hikarigaoka, Fukushima, 960-1295 Japan

**Keywords:** Rheumatoid arthritis, Interstitial lung disease, Anti-citrullinated peptide antibody, Secretory component, Rheumatoid factor, Usual interstitial pneumonia, Nonspecific interstitial pneumonia, IgA

## Abstract

**Objective:**

Rheumatoid arthritis (RA) is often complicated with chronic lung diseases (CLD), including interstitial lung disease (ILD) and airway disease, which occur as extra-articular manifestations. CLD in RA have been associated with the production of rheumatoid factor (RF), anti-citrullinated peptide antibody (ACPA), or anti-carbamylated protein (CarP) antibody. However, few validation studies have been performed thus far. In the present study, we investigated the association of RF, ACPA, and anti-CarP antibodies with RA complicated with CLD.

**Methods:**

Sera from RA patients with or without CLD were collected. The levels of serum RF, RF immunoglobulin A (IgA), ACPA IgG, ACPA IgA, and ACPA secretory component (SC) were measured using enzyme-linked immunosorbent assay.

**Results:**

The comparison of RA patients with and without CLD showed that RF IgA was associated with ILD (mean ± standard deviation: 206.6 ± 400.5 vs. 95.0 ± 523.1 U/ml, respectively, *P* = 1.13 × 10^− 8^), particularly usual interstitial pneumonia (UIP) (263.5 ± 502.0 U/ml, *P* = 1.00 × 10^− 7^). ACPA SC was associated with RA complicated with ILD (mean ± standard deviation: 8.6 ± 25.1 vs. 2.3 ± 3.4 U/ml, respectively, *P* = 0.0003), particularly nonspecific interstitial pneumonia (NSIP) (10.7 ± 31.5 U/ml, *P* = 0.0017). Anti-CarP antibodies were associated with RA complicated with ILD (0.042 ± 0.285 vs. 0.003 ± 0.011 U/ml, respectively, *P* = 1.04X10^− 11^).

**Conclusion:**

RF IgA and ACPA SC in RA were associated with UIP and NSIP, respectively, suggesting different specificities in patients with RA. Anti-CarP antibodies were associated with ILD in RA. These results may help elucidate the different pathogeneses of UIP and NSIP in RA.

**Supplementary Information:**

The online version contains supplementary material available at 10.1186/s12891-021-04985-0.

## Introduction

Rheumatoid arthritis (RA), a systemic autoimmune disease affecting synovial joints, is often complicated with chronic lung diseases (CLD), including interstitial lung disease (ILD) and airway disease (AD). RA patients complicated with ILD or AD are associated with a poor prognosis [1–5]. Thus, it is necessary to clarify the pathological conditions of ILD and AD in RA.

Rheumatoid factors (RFs) are autoantibodies against immunoglobulin G (IgG) Fc fragments; most RFs belong to the IgM class. The serum levels of RFs are linked to RA-associated ILD (RA-ILD) [6, 7]. The levels of RF IgA are also related to RA-ILD [8, 9]. Anti-citrullinated peptide antibodies (ACPAs) are autoantibodies against citrullinated peptides. Citrullinated peptides are generated through posttranslational modification of arginine residues to citrulline by peptidylarginine deiminases. The specificity of ACPA for RA is higher than that of RFs, and the serum levels of ACPA IgG are associated with RA-ILD [6, 9, 10]. Notably, idiopathic pulmonary fibrosis is associated with ACPA IgA [8, 11]. Moreover, the serum levels of ACPAs with secretory component (SC) have been linked to RA-ILD [12]. SC attaches to IgA and IgM to form secretory IgA and IgM, respectively; these secretory Igs are subsequently transported to the mucosa. Small amounts of secretory Igs have also been found in sera [13, 14]. Studies have revealed that the major portion of serum ACPA SC is composed of IgM [15]. Anti-carbamylated protein (CarP) antibodies recognize homo-citrullinated peptides generated by posttranslational modification of lysine residues. Anti-CarP antibodies were reported to be associated with ILD in RA [16]. However, few validation studies on those associations of autoantibodies with RA-ILD have been performed thus far. In the present study, we investigated the association of RF, ACPA, and anti-CarP antibodies with RA-ILD in Japan.

## Materials and methods

### Patients and sera

A total of 453 patients with RA, for whom chest computed tomography data were available, were recruited at Sagamihara National Hospital, Himeji Medical Center, Miyakonojo Medical Center, Nagoya Medical Center, and Nagasaki Medical Center from 2010 to 2017. These patients fulfilled the American College of Rheumatology criteria for RA [17] or Rheumatoid Arthritis Classification Criteria [18]. Sera from the patients were collected and analyzed for the production of autoantibodies. Based on the predominant findings of chest computed tomography, the patients were categorized as follows, usual interstitial pneumonia (UIP), nonspecific interstitial pneumonia (NSIP), AD, emphysema, or no CLD [19]. The study was reviewed and approved by the National Hospital Organization Central Institutional Review Board, Research Ethics Committees of Sagamihara National Hospital and Tokyo National Hospital. Written informed consent was provided by all the participants. This study was conducted in accordance with the principles stipulated in the Declaration of Helsinki.

### Autoantibody detection

RF was detected using an N-latex RF kit (Siemens Healthcare Diagnostics, München, Germany), and the cut-off value set by manufacturer was 15 U/ml in the kit. RF IgA was detected using a Rheumatoid factor IgA kit (Organtech Diagnostika, Mainz, Germany). Based on the 98th percentile among 52 healthy controls, the cut-off value for positivity was set to 14.572. ACPA IgG was measured using the Mesacup-2 test cyclic citrullinated peptide, and the cut-off value set by manufacturer was 4.5 U/ml in the kit (Medical & Biological Laboratories, Nagoya, Japan). ACPA IgA was detected with the Mesacup-2 test CCP and peroxidase conjugated goat anti-human IgA alpha chain antibodies (Jackson ImmunoResearch, West Grove, PA, USA). Sera and secondary antibodies were diluted (1:100 and 1:10,000, respectively) using the dilution buffer included in the kit. The levels of ACPA IgA in pooled sera from three patients with known high levels of ACPA IgA were designated to be 27 U/ml. The pooled sera were serially diluted to be used as the calibrator, and the results were presented as arbitrary units (U/ml). Based on the 98th percentile among 52 healthy controls, the cut-off value for positivity was set to 0.944. ACPA SC was detected using Immunoscan CCPlus (Svar Life Science, Malmö, Sweden) and peroxidase conjugated goat anti-human SC antibodies (Nordic-MUbio, Susteren, Netherlands). Sera and secondary antibodies were diluted (1:22 and 1:2000, respectively) using the dilution buffer included in the kit. The levels of ACPA SC in pooled sera from three patients with known high levels of ACPA SC were designated to be 27 U/ml. The pooled sera were serially diluted to be used as the calibrator, and the results were presented as arbitrary units (U/ml). Based on the 98th percentile among 52 healthy controls, the cut-off value for positivity was set to 0.966. Anti-CarP antibodies were detected using human anti-CarP ELISA kit (Wuhan Fine Biotech Co., Ltd., Wuhan, China). Sera were diluted (1:25) using the dilution buffer included in the kit. The levels of anti-CarP antibodies in the standard were designated to be 1 U/ml. The results were presented as arbitrary units (U/ml). Based on the 98th percentile among 52 healthy controls, the cut-off value for positivity was set to 0. The presence of RF and ACPA IgG autoantibodies in patients with RA has been reported in previous studies [19, 20]. Krebs von den lungen-6 (KL-6) was detected using a Picolumi KL-6 Electrochemiluminescence immunoassay system (EIDIA Co., Ltd., Tokyo, Japan) and the cut-off value set by manufacturer was 500 U/ml. Surfactant protein-D (SP-D) was detected using the SP-D kit “Yamasa” EIA II (Yamasa Corporation, Choshi, Japan) and the cut-off value for positivity set by manufacturer was 110 ng/ml. Steinbrocker stages were measured as previously described [19, 21].

### Statistical analysis

The demographic features of RA patients and autoantibody productions were compared with those of RA patients without CLD by Fisher’s exact test using 2 × 2 contingency tables or the Mann–Whitney *U* test. Receiver operator characteristic (ROC) curves for RF or ACPA were generated to compare RA patients with and without CLD. The area under the curve (AUC) values of the ROC curves with 95% confidence intervals were estimated. In addition, the optimized cut-off levels with specificities and sensitivities conditional on the highest Youden’s index were calculated. A *P* < 0.05 value denoted statistically significant difference.

## Results

### Clinical features of patients with RA

Patient characteristics are presented in Table 1. The mean age, age at onset, KL-6 levels, and SP-D levels were increased in RA patients with ILD versus those without CLD. The percentage of smokers or former smokers and KL-6 levels were higher in RA patients with AD. Furthermore, Steinbrocker stage, percentage of smokers or former smokers, KL-6 levels, and SP-D levels in RA patients with emphysema were also increased.

**Table 1:** Characteristics of patients with RA

	ILD		UIP		NSIP		AD		Emphysema		CLD(+)		CLD(−)
		*P*		*P*		*P*		*P*		*P*		*P*	
Number, n	115		46		69		112		37		264		189
Mean age, years (SD)	67.2 (8.3)	0.0009	67.1 (9.1)	0.0137	67.3 (7.8)	0.0061	65.6 (9.8)	0.0553	66.3 (8.1)	0.1247	66.4 (9.0)	0.0013	62.8 (11.1)
Male, n (%)	27 (23.5)	*0.3076	16 (34.8)	*0.0267	11 (15.9)	*0.7155	15 (13.4)	*0.2667	22 (59.5)	*1.06 × 10^−6^	64 (24.2)	*0.1668	35 (18.5)
Age at onset, years (SD)	54.1 (12.9)	0.0018	54.0 (14.3)	0.0336	54.2 (12.0)	0.0067	50.5 (14.6)	0.2257	56.5 (11.2)	0.0012	52.9 (13.6)	0.0014	49.2 (13.2)
Steinbrocker stages III and IV, n (%)	53 (46.1)	*0.0773	25 (54.3)	*0.8687	28 (40.6)	*0.0248	65 (58.6)	*0.8092	13 (35.1)	*0.0194	131 (49.8)	*0.1812	107 (56.6)
Smoker or former smoker, n (%)	42 (38.5)	*0.1186	18 (40.9)	*0.1471	24 (36.9)	*0.2730	41 (41.0)	*0.0469	28 (84.8)	*1.98 × 10^−9^	111 (45.9)	*0.0005	51 (29.0)
KL-6, U/ml (SD)	726.6 (639.4)	1.11 × 10^−15^	749.9 (652.6)	1.77 × 10^− 9^	710.5 (635.1)	3.10 × 10^−12^	383.2 (330.0)	0.0012	585.5 (466.0)	1.92 × 10^−6^	585.3 (546.4)	2.60 × 10^−13^	293.6 (294.4)
SP-D, ng/ml (SD)	122.3 (144.9)	1.95 × 10^−9^	128.6 (92.6)	2.43 × 10^− 7^	117.9 (173.8)	2.27 × 10^−6^	67.2 (70.9)	0.1588	93.2 (69.5)	0.0003	99.2 (117.4)	4.58 × 10^−7^	51.1 (40.9)

*AD* Airway disease, *CLD* Chronic lung disease, CLD(+), with CLD, CLD(−), without CLD, *KL-6* Krebs von den lungen-6, *NSIP* Nonspecific interstitial pneumonia, *RA* Rheumatoid arthritis, *SP-D* Surfactant protein-D, *UIP* Usual interstitial pneumonia

ILD group includes UIP and NSIP groups. CLD(+) group includes UIP, NSIP, AD, and emphysema groups

Data are presented as the mean value or number of each group. Standard deviations or percentages are shown in parentheses. Statistical differences were tested in comparison with the CLD(−) population by Fisher’s exact test using 2 × 2 contingency tables or the Mann–Whitney *U* test. *Fisher’s exact test

### RF, ACPA, and anti-CarP antibodies in patients with RA

**Fig. 1:**
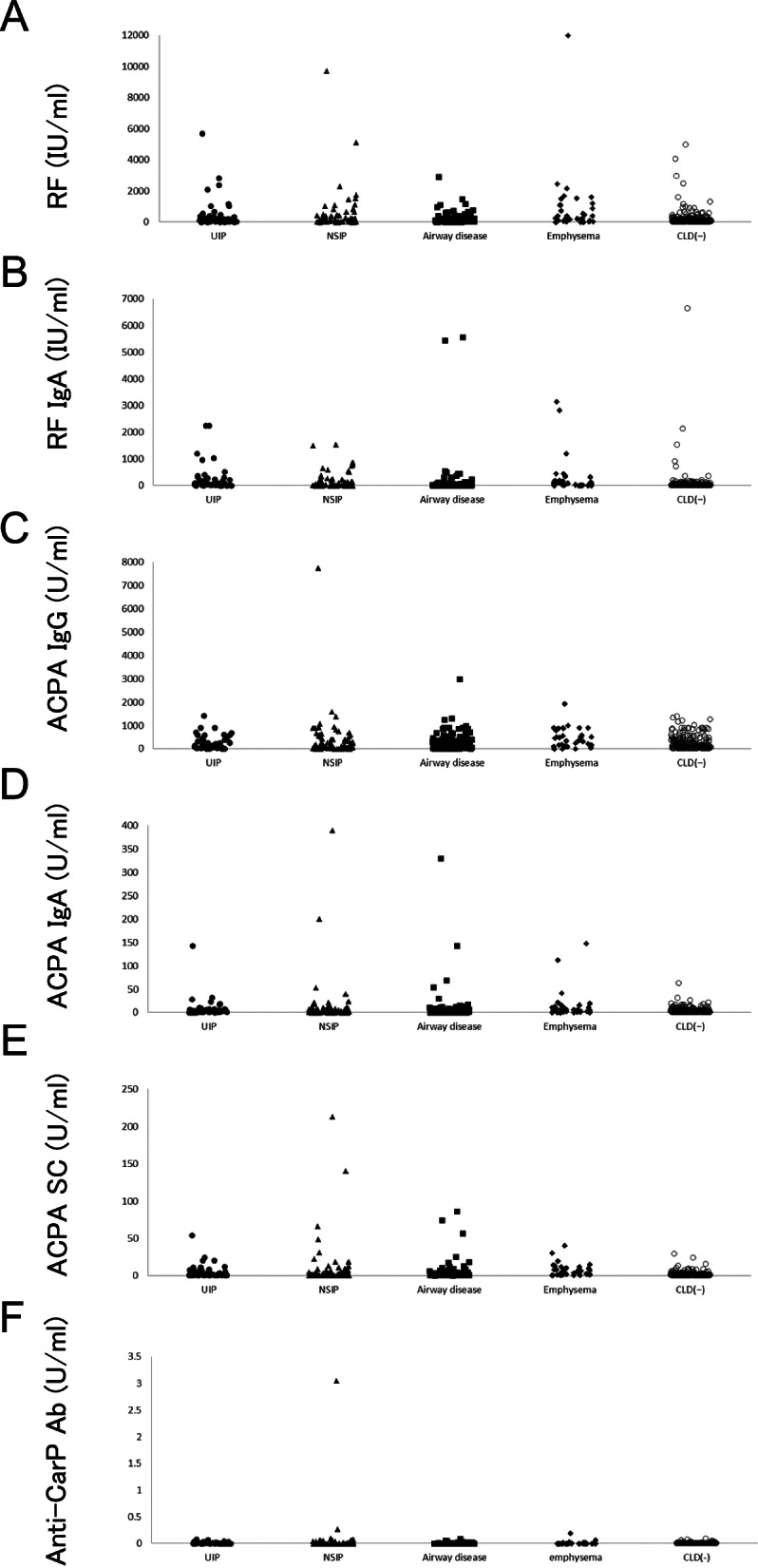
Evaluation of the RF or ACPA levels in patients with RA. Distribution of RF (**A**), RF IgA (**B**), ACPA IgG (**C**), ACPA IgA (**D**), ACPA SC (**E**), and anti-CarP Ab (**F**) levels. The filled circle, filled triangle, filled square, filled diamond, and empty circle represent RA with UIP, RA with NSIP, RA with airway disease, RA with emphysema, and RA without CLD, respectively. ACPA: anti-cyclic citrullinated peptide antibody, CLD: chronic lung disease, CLD(−): without CLD Ig immunoglobulin, NSIP: nonspecific interstitial pneumonia, RA: rheumatoid arthritis, RF: rheumatoid factor, SC: secretory component, UIP: usual interstitial pneumonia, CarP: carbamylated protein, Ab: antibody

The production of RF and ACPA was analyzed in the sera of RA patients with and without CLD (Table 2, Fig. 1). RF was associated with ILD (mean ± standard deviation: 510.9 ± 1213.6 vs. 235.69 ± 569.9 U/ml, respectively, *P* = 0.0025), AD (233.6 ± 362.6 U/ml, *P* = 0.0149), emphysema (871.4 ± 1993.6 U/ml, *P* = 0.0002), and CLD (443.8 ± 1133.3 U/ml, *P* = 9.58 × 10^− 5^). RF IgA was associated with ILD (206.6 ± 400.5 vs. 95.0 ± 523.1 U/ml, respectively, *P* = 1.13 × 10^− 8^), particularly UIP (263.5 ± 502.0 U/ml, *P* = 1.00 × 10^− 7^). RF IgA was also associated with emphysema (293.0 ± 687.4, *P* = 8.64 × 10^− 6^) and CLD (195.1 ± 601.9 U/ml, *P* = 7.21 × 10^− 7^). ACPA IgG was associated with RA with emphysema (445.6 ± 400.6 vs. 270.7 ± 308.3 U/ml, respectively, *P* = 0.0033). ACPA IgA was associated with RA with ILD (11.8 ± 42.7 vs. 4.1 ± 6.7 U/ml, respectively, *P* = 0.0159), AD (9.2 ± 34.4 U/ml, *P* = 0.0468), emphysema (14.0 ± 29.7 U/ml, *P* = 0.0020), and CLD (11.0 ± 37.6 U/ml, *P* = 0.0015). ACPA SC was associated with RA complicated with ILD (8.6 ± 25.1 vs. 2.3 ± 3.4 U/ml, respectively, *P* = 0.0003), particularly NSIP (10.7 ± 31.5 U/ml, *P* = 0.0017). ACPA SC was also associated with emphysema (7.9 ± 8.6 U/ml, *P* = 3.89 × 10^− 7^) and CLD (6.8 ± 18.7 U/ml, *P* = 3.05 × 10^− 5^). Anti-CarP antibodies were associated with RA complicated with ILD (0.042 ± 0.285 vs. 0.003 ± 0.011 U/ml, respectively, *P* = 1.04X10^− 11^). Anti-CarP antibodies were also associated with CLD (0.021 ± 0.189 U/ml, *P* = 4.75 × 10^− 5^). The positivity for RF, ACPA, and anti-CarP antibodies was also analyzed in RA patients with or without CLD (Supplementary Table S1). Although similar results were obtained, the association of the positivity of ACPA SC with NSIP in RA was not stronger than that observed for the levels of ACPA SC (Supplementary Table S1, Fig. 1E). This may be attributed to the extremely higher expression levels of ACPA SC in some RA patients with NSIP. Thus, RF IgA in RA was associated with ILD (particularly UIP), while ACPA SC in RA was associated with ILD (particularly NSIP). Additionally, anti-CarP antibodies were associated with ILD in RA.

**Table 2:** RF, ACPA, and anti-CarP Ab in patients with RA

	ILD		UIP		NSIP		AD		emphysema		CLD(+)		CLD(−)
		*P*		*P*		*P*		*P*		*P*		*P*	
RF, U/ml (SD)	510.9 (1213.6)	0.0025	501.2 (992.0)	0.0071	517.5 (1348.2)	0.0320	233.6 (362.6)	0.0149	871.4 (1993.6)	0.0002	443.8 (1133.3)	9.58X10^−5^	235.6 (569.9)
RF IgA, U/ml (SD)	206.6 (400.5)	1.13X10^−8^	263.5 (502.0)	1.00X10^−7^	168.6 (313.8)	0.0001	151.1 (731.5)	0.1945	293.0 (687.4)	8.64X10^−6^	195.1 (601.9)	7.21X10^−7^	95.0 (523.1)
ACPA IgG, U/ml (SD)	363.8 (776.1)	0.5244	282.5 (302.2)	0.5943	415.7 (962.9)	0.6334	306.4 (388.6)	0.5285	445.6 (400.6)	0.0033	350.8 (589.1)	0.1439	270.7 (308.3)
ACPA IgA, U/ml (SD)	11.8 (42.7)	0.0159	8.5 (21.4)	0.0198	14.0 (52.3)	0.1172	9.2 (34.4)	0.0468	14.0 (29.7)	0.0020	11.0 (37.6)	0.0015	4.1 (6.7)
ACPA sc, U/ml (SD)	8.6 (25.1)	0.0003	5.4 (9.3)	0.0136	10.7 (31.5)	0.0017	4.6 (12.1)	0.0999	7.9 (8.6)	3.89X10^−7^	6.8 (18.7)	3.05X10^−5^	2.3 (3.4)
Anti-CarPAb, U/ml (SD)	0.042 (0.285)	1.04X10^−11^	0.012 (0.016)	1.22X10^−9^	0.063 (0.367)	1.02X10^−7^	0.004 (0.012)	0.7585	0.010 (0.032)	0.1229	0.021 (0.189)	4.75X10^−5^	0.003 (0.011)

*ACPA* Anti-citrullinated peptide antibody, *AD* Airway disease, *CLD* Chronic lung disease, CLD(+) with CLD, CLD(−) Without CLD, *ILD* Interstitial lung disease, *NSIP*, Nonspecific interstitial pneumonia, *RF* Rheumatoid factor, *RA* Rheumatoid arthritis, *SC* Secretory component, *UIP* Usual interstitial pneumonia

The ILD group includes the UIP and NSIP groups. The CLD(+) group includes the UIP, NSIP, AD, and emphysema groups

Data are presented as the mean value of each group; standard deviations are shown in parentheses

Statistical difference was tested in comparison with the CLD(−) population using the Mann–Whitney *U* test

ROC curves for RF, ACPA, and anti-CarP antibodies were generated to compare RA patients with and without CLD (Supplementary Fig. S1). The AUC values of the ROC curves with 95% confidence intervals were calculated. However, AUC values of these ROC curves were < 0.7. These data indicated that RF, ACPA, and anti-CarP antibodies are not sufficiently strong biomarkers for the diagnosis of CLD.

## Discussion

In the present study, RF IgA was associated with RA-ILD (particularly UIP), while ACPA SC was associated with RA complicated with ILD (particularly NSIP). Anti-CarP antibodies were associated with ILD in RA. The association of RF IgA with RA-ILD was previously reported [8, 9]. Although this association was confirmed in this study, the stronger association with UIP was not observed. The association of ACPA SC with RA-ILD was also previously reported [12], and a stronger association with NSIP was found in the present study. Thus, the present results suggested different specificities of RF IgA for UIP and ACPA SC for NSIP in patients with RA. Furthermore, the evidence suggests the involvement of these autoantibodies in the development of UIP or NSIP in RA.

The data obtained from this study indicates that RF, ACPA, and anti-CarP antibodies are not good biomarkers for the diagnosis of ILD or CLD compared with the levels of KL-6 or SP-D (Tables 1 and 2, Supplementary Fig. S1). However, the association of RF IgA with UIP may elucidate the pathogenesis of UIP in RA. Analogically, the association of ACPA SC with NSIP in RA may explain the pathophysiology of NSIP in RA. Autoantibody levels in RA with AD were lower (Table 2), suggesting the heterogeneity of CLD in RA. In contrast, the expression levels of RF and ACPA were elevated in RA patients with emphysema; notably, the percentages of males and ever smokers were higher in this group of patients than in other groups. It is established that smoking increases the expression of peptidylarginine deiminase 2 and generates citrullinated autoantigens in the lung [22, 23]. These data suggest that smoking affects the autoantibody production and the development of RA; nevertheless, it was difficult to determine the roles of autoantibodies in the development of emphysema in RA. The origin of ACPA SC remains unknown and may be the lung, gastrointestinal tract, or oral cavity. In RA patients with emphysema, the origin of these antibodies may be the lung. CarPs were detected in the synovial tissues of RA patients and lung tissues from smokers [24, 25]. Although anti-CarP antibodies were not increased in RA with emphysema (Table 2), they might react with homo-citrullinated peptides in lung and causes inflammatory responses leading to ILD, but not to emphysema. Some studies reported on the associations of autoantibody productions and RA-ILD, namely anti-citrullinated alpha-enolase peptide-1 antibodies [26, 27], anti-citrullinated heat shock protein 90 antibodies [28], and anti-malondialdehyde-acetaldehyde antibodies [29]. These results suggested the involvement of several autoantibodies on the pathogenesis of ILD in RA. Epitope spread against citrullinated peptides may contribute to the development of RA and RA-ILD [30, 31]; the citrullinated autoantigens of ACPA SC in RA-ILD should be validated in future investigations.

To the best of our knowledge, this is the first study to report the different specificities of RF IgA for UIP and ACPA SC for NSIP in patients with RA. The sample size of the present study was small. Therefore, additional large-scale studies on RF IgA for UIP and ACPA SC for NSIP should be conducted to validate the present findings. Serum autoantibodies with SC have also been detected in other collagen vascular disease-associated ILD than RA. Serum anti-proteinase 3 autoantibodies with SC were detected in patients with anti-neutrophil cytoplasmic antibody-associated vasculitis [32]. Autoantibody profiles in patients with other collagen vascular disease-associated ILD than RA or idiopathic interstitial pneumonia were not analyzed in the present study. Future investigations should analyse RF IgA, ACPA SC, and anti-CarP antibodies in these patients.

## Supplementary Information

**Additional file 1.**

**Additional file 2.**
