# Supplementary material for: Serum rheumatoid factor IgA, anti-citrullinated peptide antibodies with secretory components, and anti-carbamylated protein antibodies associate with interstitial lung disease in rheumatoid arthritis
Source: BMC Musculoskelet Disord. 2022 Jan 13;23:46. doi: 10.1186/s12891-021-04985-0 (PMC8756729; doi:10.1186/s12891-021-04985-0)
Supplement: Supplementary file 1 — Additional file 1. [file 12891_2021_4985_MOESM1_ESM.pdf]

Supplementary Table S1. The positivity of RF, ACPA, and anti-CarP Ab in the RA patients.

	ILD		UIP		NSIP		AD		Emphysema		CLD(+)		CLD(−)
		P		*P*		*P*		*P*		*P*		*P*	
RF positive, n (%)	104 (90.4)	0.0467	41 (89.1)	0.2765	63 (91.3)	0.0817	103 (92.0)	0.0173	32 (86.5)	0.6381	239 (90.5)	0.0072	154 (81.5)
RF IgA positive, n (%)	80 (69.6)	$1.96X10^{-5}$	38 (82.6)	$2.47X10^{-6}$	42 (60.9)	0.0242	62 (55.4)	0.0742	30 (81.1)	$5.02X10^{-5}$	172 (65.2)	$1.45X10^{-5}$	84 (44.4)
ACPA IgG positive, n (%)	105 (91.3)	0.8319	43 (93.5)	1.0000	62 (89.9)	0.6162	102 (91.1)	0.8299	34 (91.9)	1.0000	241 (91.3)	0.8641	174 (92.1)
ACPA IgA positive, n (%)	85 (73.9)	0.0247	35 (76.1)	0.0607	50 (72.5)	0.1069	84 (75.0)	0.0164	28 (75.7)	0.0962	197 (74.6)	0.0020	115 (60.8)
ACPA SC positive, n (%)	75 (65.2)	0.0090	31 (67.4)	0.0335	44 (63.8)	0.0494	64 (57.1)	0.2336	31 (83.8)	0.0001	170 (64.4)	0.0020	94 (49.7)
Anti-CarPAb positive, n (%)	63 (54.8)	$8.70X10^{-10}$	29 (63.0)	$6.41X10^{-8}$	34 (49.3)	$8.43X10^{-6}$	20 (17.9)	0.6540	11 (29.7)	0.1967	94 (35.6)	0.0004	38 (20.1)

ILD group includes UIP and NSIP groups. CLD(+) group includes UIP, NSIP, AD, and emphysema groups. Number of each group is shown. Percentages are shown in parenthesis. Difference was tested in the comparison with the CLD(−) population by Fisher's exact test using 2×2 contingency tables. ACPA, anti-citrullinated peptide antibody; AD, airway disease; CLD, chronic lung disease; CLD(+), with CLD; CLD(−), without CLD; ILD, interstitial lung disease; NSIP, nonspecific interstitial pneumonia; RA, rheumatoid arthritis; RF, rheumatoid factor; SC, secretory component UIP, usual interstitial pneumonia, CarP; carbamylated protein, Ab; antibody.
