# Supplementary material for: Serum rheumatoid factor IgA, anti-citrullinated peptide antibodies with secretory components, and anti-carbamylated protein antibodies associate with interstitial lung disease in rheumatoid arthritis
Source: BMC Musculoskelet Disord. 2022 Jan 13;23:46. doi: 10.1186/s12891-021-04985-0 (PMC8756729; doi:10.1186/s12891-021-04985-0)
Supplement: Supplementary file 2 — Additional file 2. [file 12891_2021_4985_MOESM2_ESM.pdf]

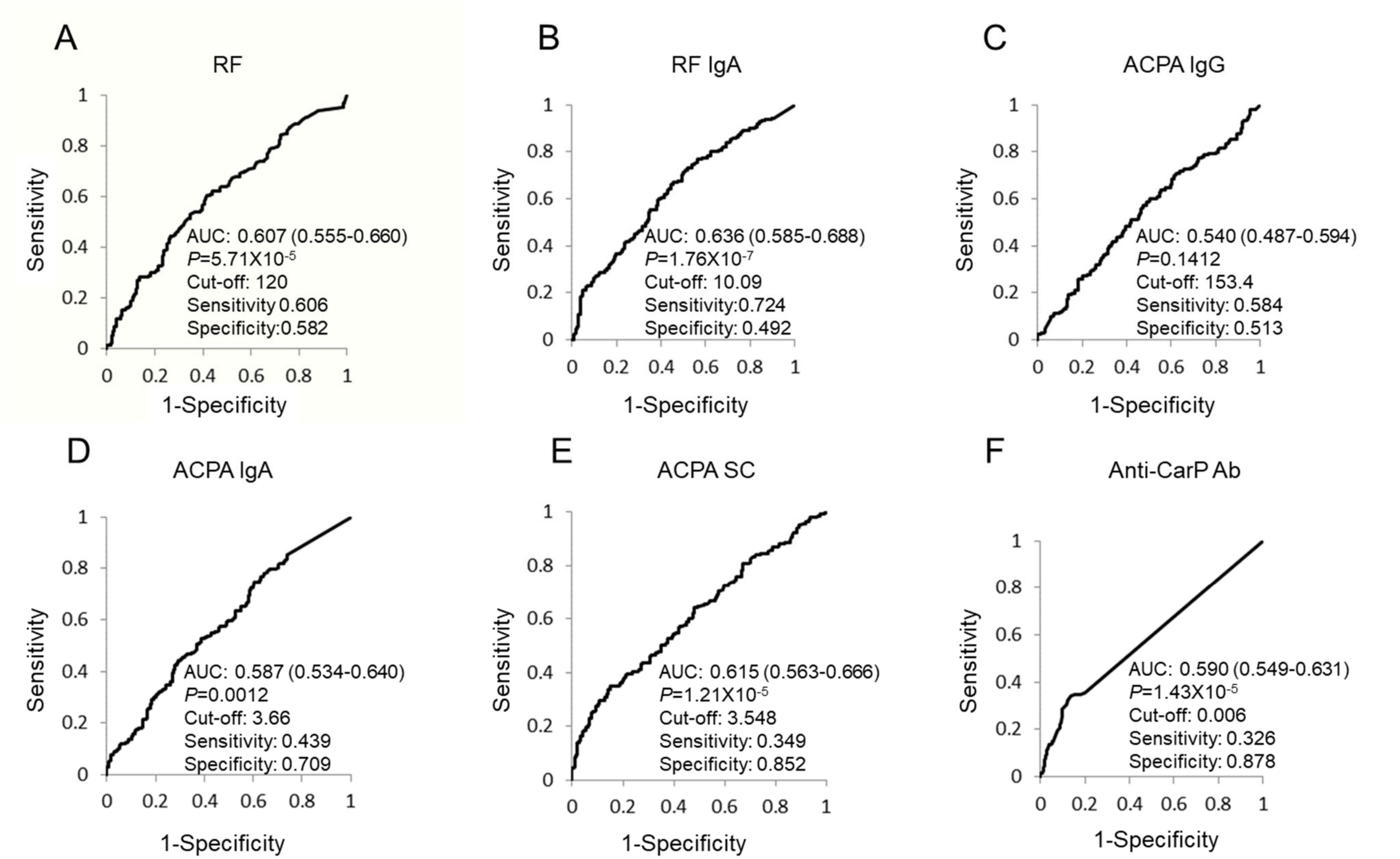

Supplementary Figure S1. Receiver operating characteristic (ROC) curves using RF, ACPA, and anti-CarP Ab in the comparison between CLD(+) and CLD(−) RA. ROC curves for RF (A), RF IgA (B), ACPA IgG (C), ACPA IgA (D), ACPA SC (E), and anti-CarP Ab (F) were generated in the comparison between CLD(+) and CLD(−) RA. The area under the curve (AUC) values of the ROC curves with 95% confidence intervals and the optimized cut-off levels with specificities and sensitivities are described. ACPA, anti-cyclic citrullinated peptide antibody; AUC, area under the curve; CLD, chronic lung disease; CLD(+), with CLD; CLD(−), without CLD; Ig immunoglobulin; RF, rheumatoid factor; ROC, receiver operating characteristic; SC, secretory component, CarP, carbamylated protein; Ab, antibody.
